# Landscape of Research Areas for Zeolites and Metal-Organic Frameworks Using Computational Classification Based on Citation Networks

**DOI:** 10.3390/ma10121428

**Published:** 2017-12-14

**Authors:** Takaya Ogawa, Kenta Iyoki, Tomohiro Fukushima, Yuya Kajikawa

**Affiliations:** 1 Department of Chemistry, Massachusetts Institute of Technology, 77 Massachusetts Avenue, Cambridge, MA 02139, USA; 2 Department of Chemical Engineering, Massachusetts Institute of Technology, 77 Massachusetts Avenue, Cambridge, MA 02139, USA; 3 School of Environment and Society, Tokyo Institute of Technology, 3-3-6 Shibaura, Minato-ku, Tokyo 108-0023, Japan; kajikawa@mot.titech.ac.jp

**Keywords:** research and development management, citation network analysis, bibliometrics, porous materials, zeolites, metal-organic frameworks

## Abstract

The field of porous materials is widely spreading nowadays, and researchers need to read tremendous numbers of papers to obtain a “bird’s eye” view of a given research area. However, it is difficult for researchers to obtain an objective database based on statistical data without any relation to subjective knowledge related to individual research interests. Here, citation network analysis was applied for a comparative analysis of the research areas for zeolites and metal-organic frameworks as examples for porous materials. The statistical and objective data contributed to the analysis of: (1) the computational screening of research areas; (2) classification of research stages to a certain domain; (3) “well-cited” research areas; and (4) research area preferences of specific countries. Moreover, we proposed a methodology to assist researchers to gain potential research ideas by reviewing related research areas, which is based on the detection of unfocused ideas in one area but focused in the other area by a bibliometric approach.

## 1. Introduction

**Scheme 1:**
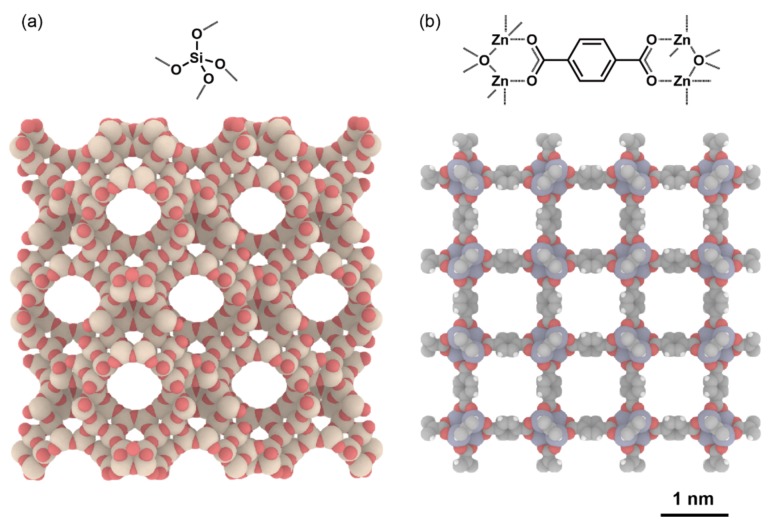
Molecular level structure (top) and porous structure (bottom) of (**a**) FAU zeolite and (**b**) MOF-5.

The field of porous materials has been investigated for a very long time and is still very attractive to researchers in chemistry, physics, biology, and material sciences. In particular, zeolites and metal-organic frameworks (MOFs) are representative examples of microporous crystalline materials (Scheme 1).

Zeolites are crystalline microporous metal oxide-based materials. The structures of zeolite consist of tetrahedral TO_4/2_ (T = Si, Al, Zn, P, Ga, Ge, B, Be, etc.) primary building units, where each oxygen atom is connected to two tetrahedral atoms. The first zeolite was discovered in 1756 by a mineralogist [1]. Later, in the 1940s, zeolites started to be investigated in academic research areas [2]. Zeolites are used for widespread applications in many fundamental industrial processes related to ion-exchange, catalysis, adsorption, and so on.

MOFs are organic–inorganic hybrid materials with periodic porous structures. The structures of MOFs consist of coordination bonds between metal ions and organic ligands [3]. Even though MOFs have long been known as a class of coordination compounds such as Prussian blue analogues [4], the number of reports on these dramatically increased after a demonstration of permanent porosity [5]. A designable porous structure of MOFs can be utilized for gas storage, separation, and transport-based applications [6,7].

Researchers need to read tremendous numbers of papers to gain a “bird’s eye” view of their research area. More than 67,000 and 23,000 research papers have been published as of March 2016 on zeolites and MOFs, respectively. In addition, these research areas are still expanding; over 5000 papers were published on zeolites and MOFs in 2015 (vide infra). Individual researchers need to perform “manual” classification of each article to review even a single research area, which is a time-consuming task. Reading reviews of research areas are very effective to understand whole trends of the research; however, common reviews are written by individual researchers and have to be subjective to their knowledge and interests. It can lead to a biased overview that misleads not only scientists but government agencies when they determine budgets for research. An objective landscape of whole research areas is difficult to obtain in these ways.

In principle, zeolites and MOFs are both classified as crystalline microporous materials. We hypothesized that the research approach for one area could be simply adopted in other similar research areas; the approach that has been used for zeolites could be applied to the research on MOFs, and vice versa. Bridging these two research areas has the potential to develop each area by applying the knowledge accumulated in these areas. This “knowledge”-based research requires a well-surveyed database that includes (1) what has been studied as research topics; (2) whether they are major research topics; and (3) what can be applied from one research area to another. Traditional approaches such as the manual reading of papers by individual researchers are not effective at bridging even similar areas.

In order to solve the aforementioned problems, bibliometrics is a powerful tool for obtaining an objective overview of scientific activities in a manner that individual cannot handle. Notably, citation network analysis of papers is being used effectively to identify emerging academic research clusters and analyze their characteristics without reading individual papers [8,9,10]. Researchers in bibliometrics have studied effective methodology to illustrate an overview of research fields and detect emerging research with text-based and citation-based methodologies. Citation-based clustering works better than a text-based one and can extract semantic coherent clusters [11]. There are three different methods to create a citation network, i.e., direct citation, co-citation, and bibliographic coupling. In the previous literature, a direct citation is shown to be best-suited to identify emerging clusters [12,13]. Citation network analysis has been used for the classification of various research areas in subcategories [14,15,16,17,18,19,20]. It detects characteristics of subcategory such as the average publication years, number of publications, and country distribution in terms of publication numbers, which can be utilized for the evaluation of subcategories. For example, the average publication years and the number of publications in a classified cluster can detect which research area is fresh and not large, i.e., emerging research area. In addition, researchers generally tend to be surrounded by domestic information and focus on the hot topic in their country, which could be not important in main stream in the whole science communities. This can be avoided by the information about country distributions of publication number. Moreover, the information can be important objective data not only for scientists but also for government agencies to know the strength of their countries and determine the budget ratio for research area. This methodology is not limited to academic journals but can also be used for patent data [21]. This analytical technique is also useful for making comparisons between different research areas based on their characteristics [22]. Comprehensive analysis and comparisons can highlight unfocused topics, which have the potential to emerge in the future [23,24,25].

**Scheme 2:**
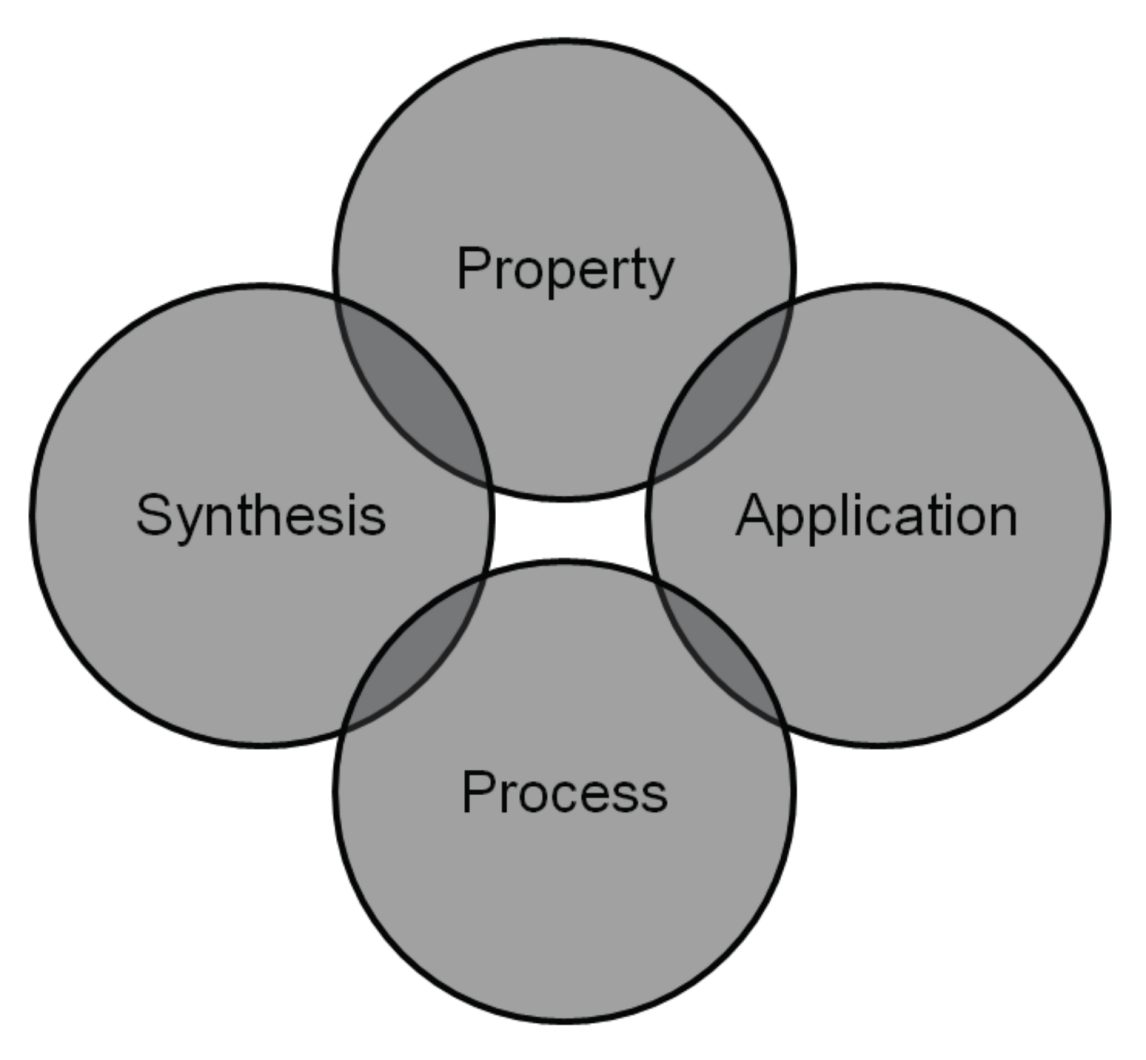
Stages of material research.

In this study, citation network analysis was applied for clustering the publications on zeolites and MOFs into groups that focused on specific research areas, which contribute to a comprehensive and objective understanding of each area. These clusters were also classified using four stages to evaluate the maturity of zeolites and MOFs. It is well-recognized that material research has some stages for the research topics [26,27,28]. These stages are defined as synthesis, property, process, and application (see Scheme 2). First, a material study focuses on “synthesis”, in which a new material is proposed. The research then moves to the “process” or “property” stage, which focuses on efficient methods to synthesize and investigate properties of a synthesized material, respectively. Finally, the research reaches the “application” stage, which applies a synthesized material for practical use. Although each stage is linked to the next or previous level, a research area that focuses on synthesis rarely examines applications at the same time, and vice versa. Moreover, trends of publications have been analyzed by focusing on which journals accept each research area. This would give us high impact areas of research to be focused on. Countries of institutes which perform each research in publications have been investigated to detect research preference of countries. This analysis will be important to be objective for whole research areas because researchers easily misunderstand research trends by surrounding domestic information. The objective data based on our methods should be beneficial to avoid misleading the landscape of research areas. Furthermore, we proposed several novel research areas that have not been given much attention based on the analyzed research areas. This approach can be a method to find future research areas by analyzing similar research areas using bibliometrics.

## 2. Data and Methods

### 2.1. Data

We collected bibliographic data from academic publications on zeolites and MOFs. The academic papers, including the title, author, publication year, abstract, address, and references, were retrieved from the Science Citation Index Expanded (SCI-EXPANDED), compiled by the Thomson Reuters Institute for Scientific Information (ISI). We used the query “zeolit*” to collect data on zeolites, which could include “zeolite*” and “zeolitic*”. In the case of MOFs, we used a rather complex query to identify and recall relevant papers. The query was as follows: “metal-organic framework*” or “porous coordination polymer*” or “*mof-*” or “zeolitic imidazolate framework*” or “zif-*” or “mil-*” or “covalent organic framework*” or “cof-*”. The total number of publications on MOFs did not change by more than 100, even when one phrase was removed. It means that other additional terms hardly change the total number and the employed query is sufficient to analyze the whole MOF research area. Zeolitic imidazolate frameworks (ZIF) can be classified into both categories from these queries. It should be included in MOF research areas due to its definition, and therefore, manually removed in this analysis. Even though COFs and MOFs are different based on the classification for coordination compounds and organic compounds, the important property between them are similar in principle: porous materials and molecular components are tunable. Therefore, the research areas on COFs have been analyzed as a part of MOFs. Data collection was carried out in March 2016.

### 2.2. Methods

**Scheme 3:**
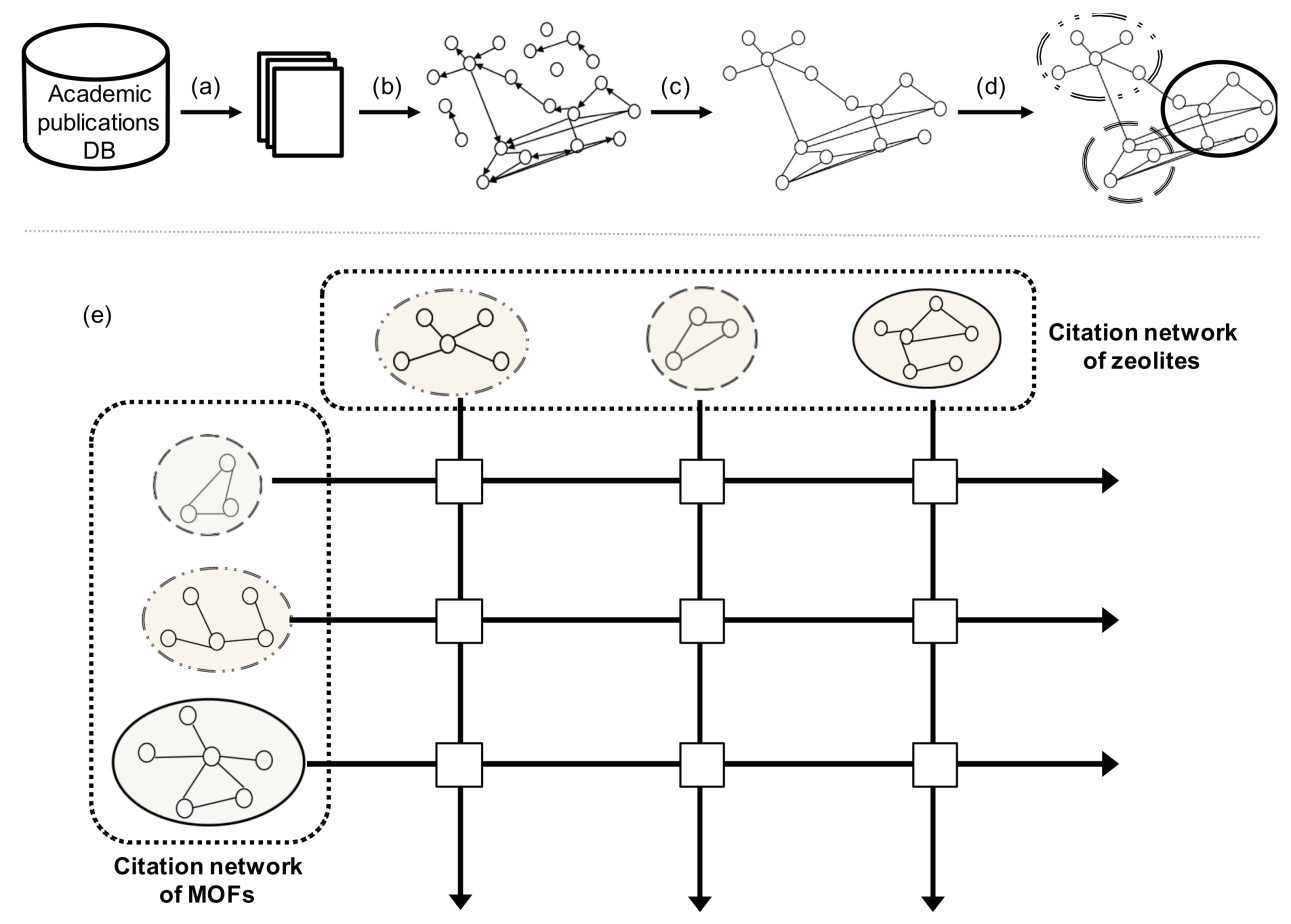
Schematic illustration of the clustering process: (**a**) Data collection; (**b**) papers as nodes and citations as link; (**c**) extraction of important clusters; (**d**) topological clustering; (**e**) Examination by experts and comparisons between MOF and zeolites clusters (in terms of publication number, average publication years, country distribution of publication number, etc.) and suggestion of researches expected to be developed in future.

Our analytical procedure is schematically shown in Scheme 3. In step (a), the data from academic papers were downloaded. In step (b), we constructed citation networks by treating the papers as nodes and the citations as links. According to a previous study, intercitation, which is also known as direct citation, is the best-demonstrated approach for detecting emerging trends [13]. In the network analysis, only the data for the largest graph component were used; we eliminated data not linked to any other papers in step (c). After extracting the largest connected component, in step (d), the network was divided into clusters using the topological clustering method of Newman’s algorithm, which extracts tightly knit groups of nodes [29,30,31]. Newman’s algorithm employs the following equation:

(1) $$Q=\overset{M}{\underset{s=1}{\sum }}\left[\frac{l_{s}}{l}-{\left(\frac{d}{2l}\right)}^{2}\right]$$

where *Q* is the independence of the module, *M* is the number of clusters, *s* is the given cluster, *l* is the number of links in the whole network, *l_s_* is the number of links between both nodes within cluster *s*, and *d_s_* is the sum of the links of the nodes in cluster *s*. Equation (1) means the sum of “the probability that reference links exists within the cluster *s*, subtracted by the probability of random link”. In Newman’s algorithm, the clusters are divided into subgroups to maximize *Q*. Starting with a state in which each node is the only member in one of the *M* clusters, we repeatedly join clusters together in pairs by choosing at each step the join that results in the largest increase in *Q*. During the iteration, *Q* gradually increases and after reaching the maximum point, it rapidly decreases [32]. To obtain the maximum *Q*, we stopped the iterations when the change of *Q* becomes negative. These procedures are inclined to create clusters based on well-cited publication. This algorithm identifies well-separated clusters in the research area. These four steps were applied to whole publication data (more than 67,000 and 23,000 papers for zeolites and MOFs, respectively). Finally, in step (e), all the cluster was examined and compared by two experts with PhDs in the research area of zeolites (K.I.) or MOFs (T.F.). The research topic in a cluster was classified by the experts. The classification into four groups (Scheme 2) was also performed by the experts. We showed objective data in the Results section and discussed in Discussion section to extract some insights from the data.

High impact research areas were examined using a high impact journal (HIJ) index of a cluster related to the impact factor of the journal published as of March 2016. Contrary to citation network analysis, where well-cited papers are important, impact factor is also important because some influential papers are not cited for many times if the number of researchers in the field is small whereas the clustering process was based on citation network, which means a well-cited paper is important. HIJ should be suitable to detect high impact areas irrelevant to the number of researchers. We focused on HIJs (Nature, Nature Chemistry, Nature Materials, Science, Proceedings of National Academy of Sciences of the United States of America, Journal of the American Chemical Society, Angewandte Chemie International Edition, Chemical Science, Energy Environmental Science, Advanced Materials, and Chemical Communications) (see Supplementary Materials for total number of publication). The index was calculated according to Equation (2), using the impact factors of March 2016 for each domain for zeolites and MOFs. Following equation means that averaged value of impact factor of research areas.

(2) $$High\;impact\;journal\;index\;of\;a\;cluster=\;\frac{\sum^{\text{​}}n_{ij}\times IF_{j}}{n_{total}}$$

where *IF_j_*, *i, n_i,j_*, and *n_total_* are the impact factors of journals in 2016, a cluster, the number of publications of journal *j* in cluster *i*, and the number of total publications in each area (MOFs or zeolites) respectively (see SI for further information).

## 3. Results

Citation network analysis was applied for zeolites and MOFs. We obtained an overview by focusing on the research area, publication year, and publication country.

### 3.1. Number of Publications on Zeolites and MOFs

**Figure 1:**
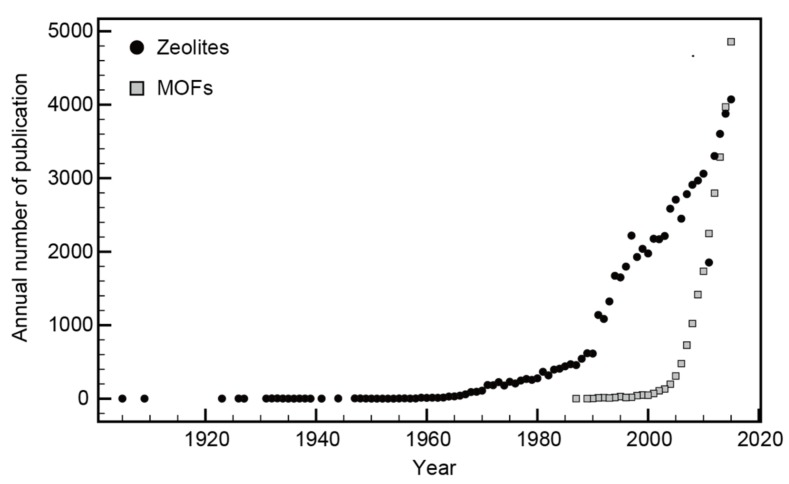
Number of publications on zeolites (black circles) and MOFs (gray squares) in given year.

First, we investigated the total number of publications on zeolites and MOFs for given years, as shown in Figure 1. The growth of zeolite publications could be divided into three stages: (i) 1950–1970; (ii) 1970–2000; and (iii) 2000 to the present. The growth of zeolite research is becoming slower: the number of annual reports doubled in (i) 2.6 years; (ii) 7 years; and (iii) 17.8 years. The growth of MOFs publications could be fitted in single exponential fashion, with the number of reports doubling every 2.3 years for MOFs. These large numbers of reports, 67,000 and 23,000 papers for zeolites and MOFs, respectively, and their rates of increase justify the importance of the overview based on bibliometrics in this report with respect to each aspect, as discussed later.

### 3.2. Populations and Representative Researchers in Research Countries 

**Figure 2:**
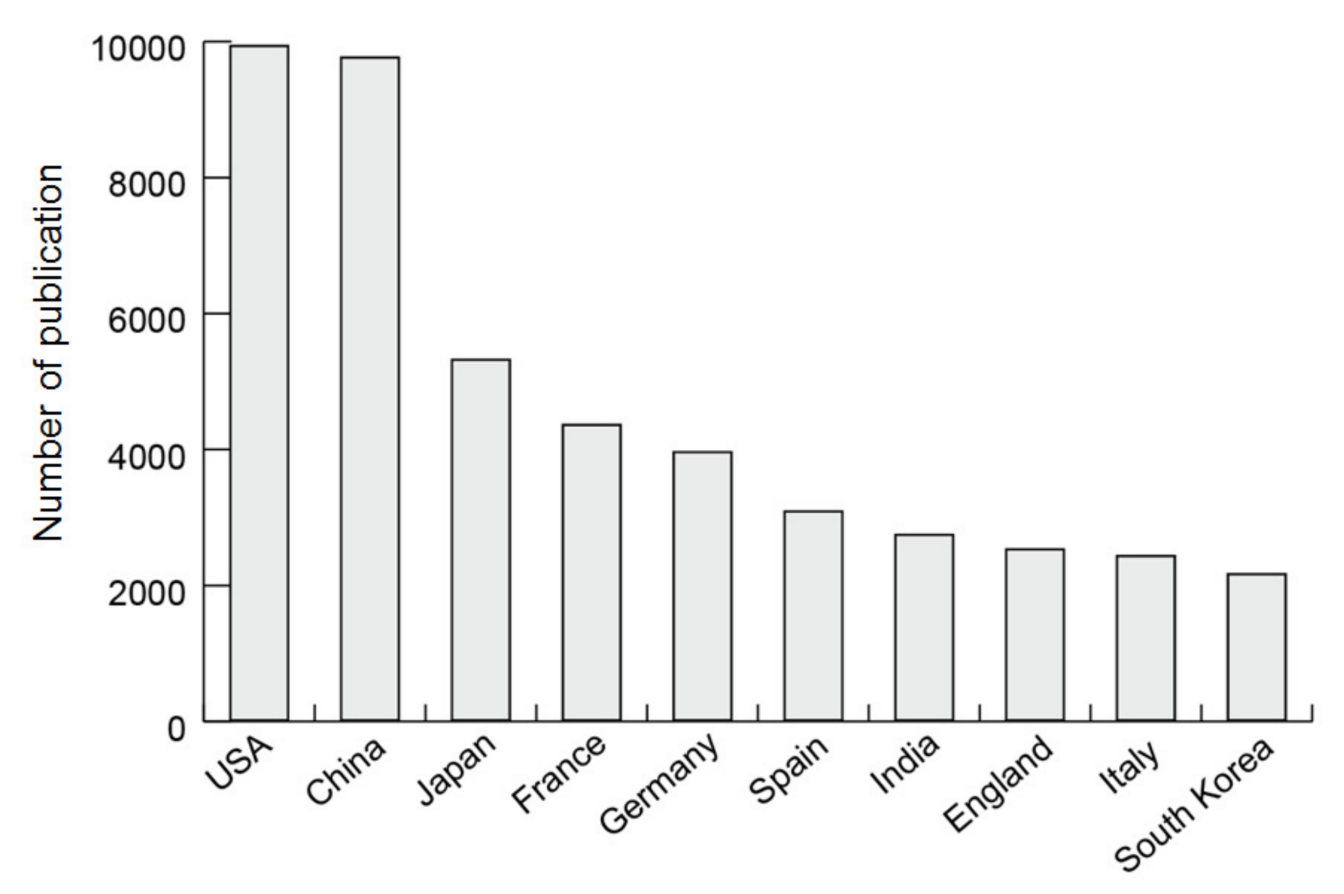
Number of publications from each country in zeolite research area.

**Figure 3:**
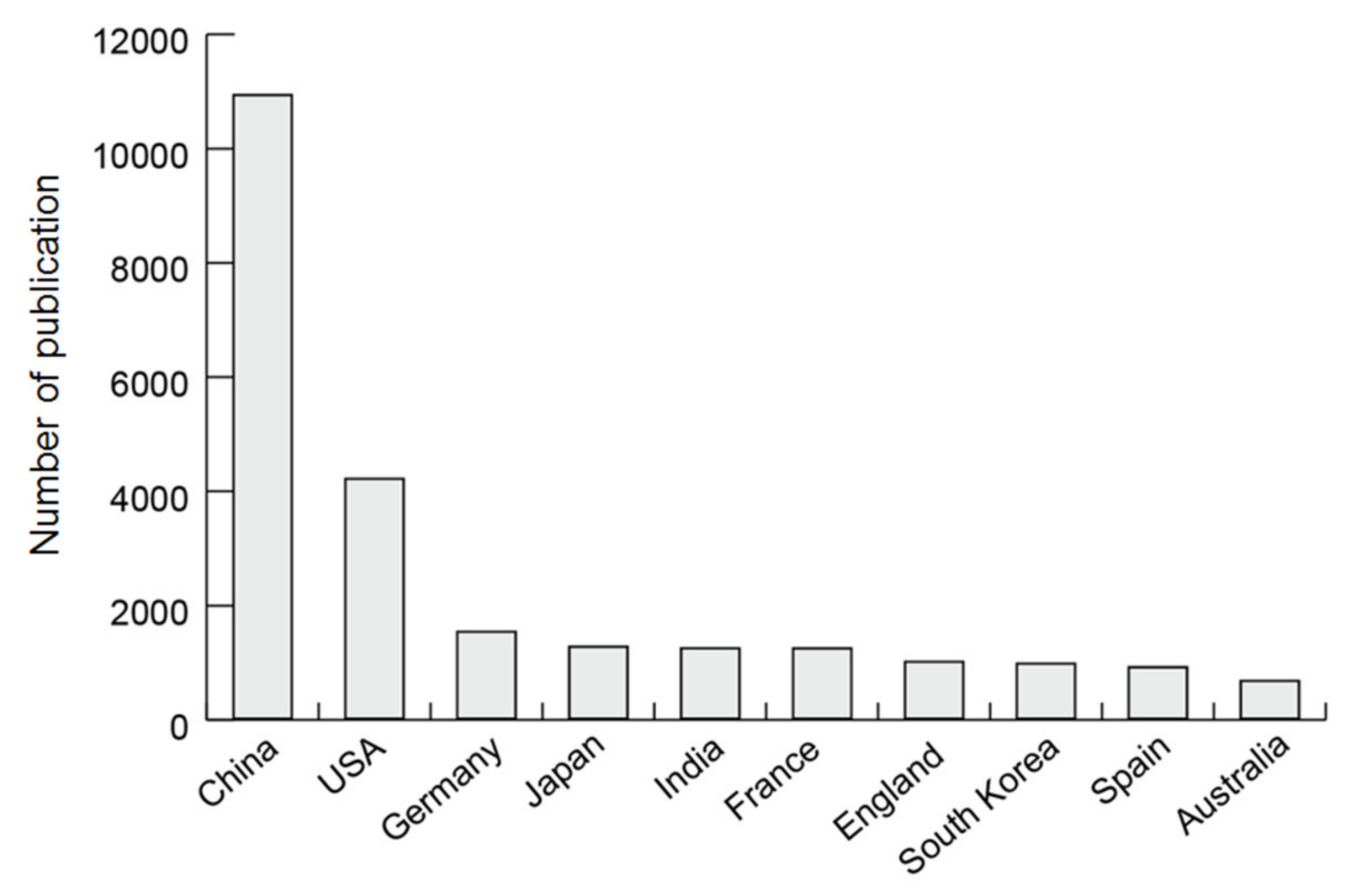
Number of publications from each country in MOF research area.

The research countries are different for zeolites and MOFs, as shown in Figure 2 and Figure 3. Zeolites have been studied the most in the US and China, with distributed studies in the European region. On the other hand, MOFs have mostly been studied in China and the US, which occupy 50% of the total research area. Research areas are heavily dependent on the researchers in given countries, as discussed later.

### 3.3. Research Area Population

#### 3.3.1. Research Area Population for Zeolites

**Figure 4:**
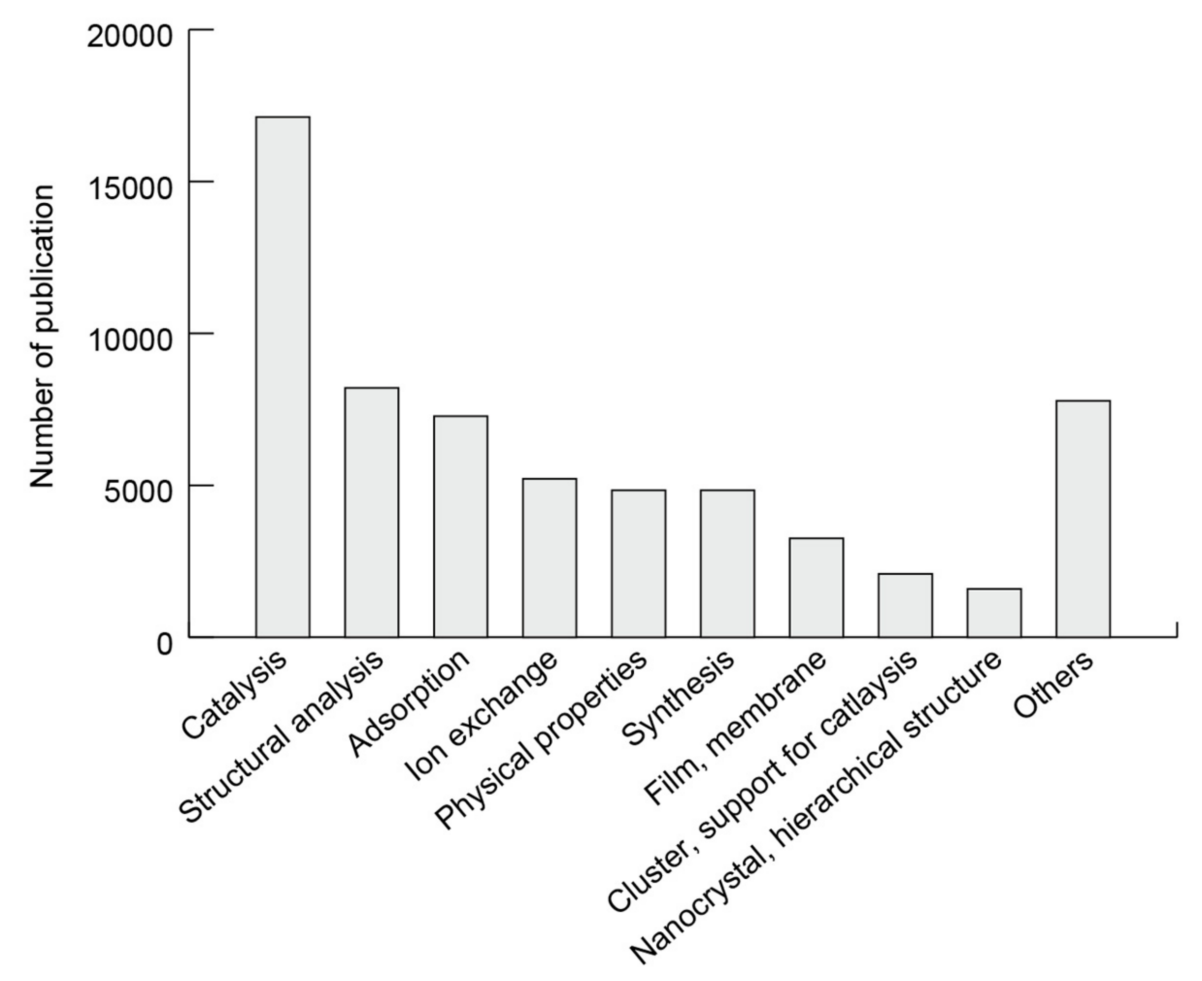
Research areas for zeolites from citation network analysis.

The population of research categories for zeolites is shown in Figure 4. These include the following: (1) catalysis (~17,000 papers, 26%); (2) structural analysis (~8000 papers, 13%); (3) adsorption (~7000 papers, 11%); (4) ion-exchange (~5000 papers, 8%); (5) physical properties (~5000 papers, 7%); (6) synthesis (~5000 papers, 7%); (7) film and membrane (~3000 papers, 5%); (8) support for metal clusters (~2000 papers, 3%); and (9) nanocrystals and hierarchical porosities (~1500 papers, 2%).

We will briefly explain these research areas and the major subcategories. Category (1) catalysis includes many types of catalytic applications using zeolites, such as cracking, reduction–oxidation (redox), and isomerization. Both zeolite framework metals and extra-framework metal cations are studied and used as active sites [33,34]. Category (2) structural analysis includes characterizations of zeolites using spectroscopies and microscopes [35]. Two large subcategories in (3) adsorption are diffusion studies of various molecules through zeolitic micropores [36,37] and the removal of toxic volatile organic compounds [38]. Category (4) ion-exchange includes the cation exchange properties of zeolites and locations of exchanged cations [39]. Category (5), physical properties, considers the optical, electrical, and other properties of zeolites and guests encapsulated in the pores [40]. Category (6) synthesis mainly considers how zeolites are synthesized using hydrothermal treatments. Other methods for efficient synthesis and the synthesis of new structures are also reported [41,42]. Category (7), the film and membrane category, considers how zeolite films and membranes are fabricated for separating small molecules such as water/ethanol [43,44]. Zeolites can also be used as supports for metal clusters, as categorized in category (8), clusters and supports for catalysis [45]. Category (9), the nanocrystal and hierarchical structure category considers the synthesis of zeolites with small crystal sizes and/or larger pores (called mesopores and macropores) in addition to micropores [46].

#### 3.3.2. Research Area Population for MOFs

**Figure 5:**
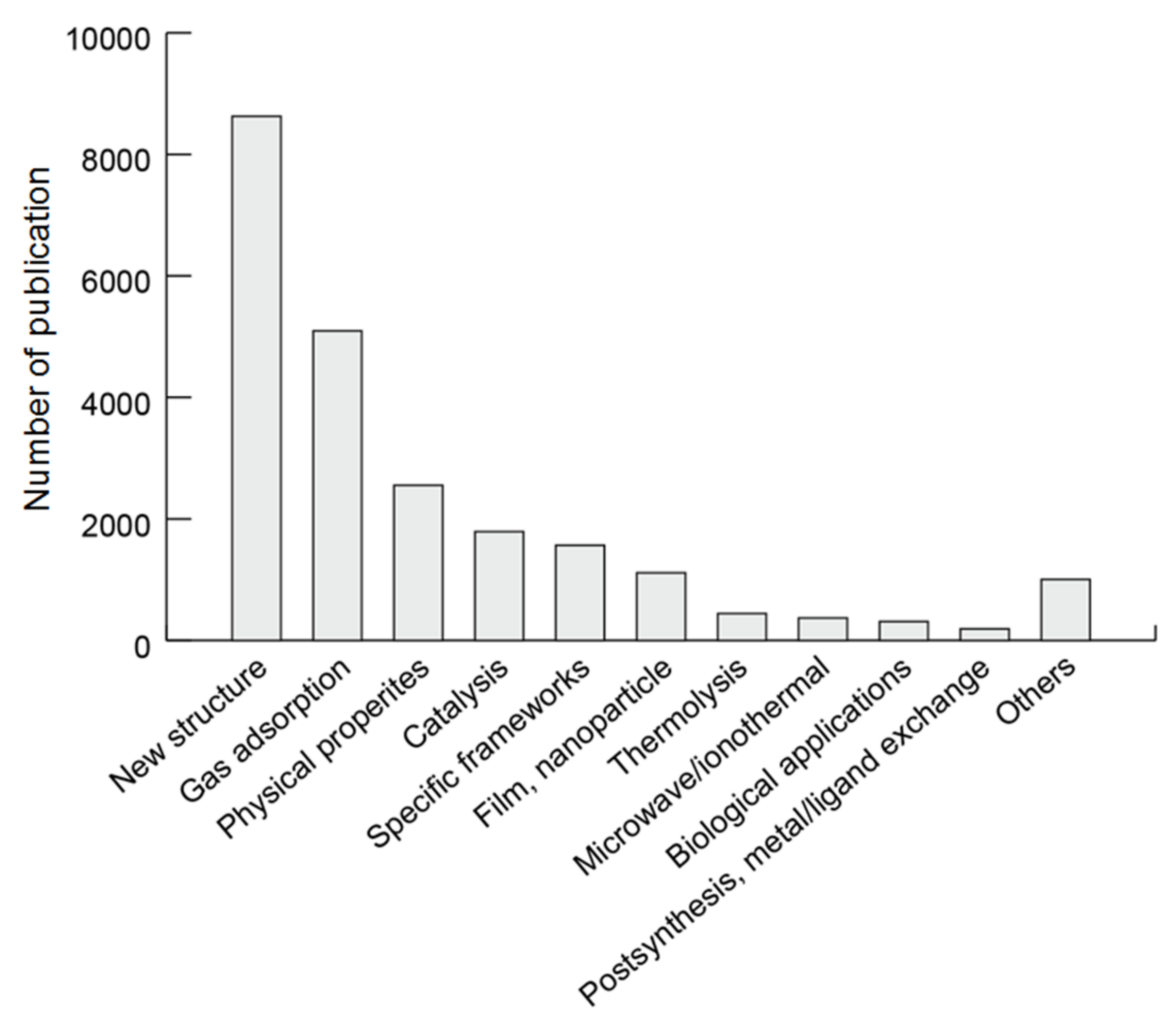
Research areas for MOFs from citation network analysis.

In the cluster analysis, we analyzed the population of research categories for MOFs, as shown in Figure 5. These include (1) the syntheses of new MOFs and their crystallography (~8600 papers, 37%); (2) properties that are related to gas adsorption (~5000 papers, 22%); (3) physicochemical properties (~2500 papers, 11%); (4) catalysis (~1700 papers, 8%); (5) field for specific frameworks (~1500 papers, 7%); (6) MOF films and nanoparticles (~1100 papers, 5%); (7) thermolysis (400 papers, 2%); (8) microwave and ionothermal syntheses (~360 papers, 1%); (9) bioapplications (~300 papers, 1%); and (10) postsynthesis and metal/ligand exchange(~200 papers 1%).

Briefly, we will explain the major subcategories for each research area. Category (1), the synthesis of new MOFs, includes a variety of materials with new structures composed of different building units, along with the topology of materials [47]. Most of these were fundamental synthetic studies involving hydrothermal reaction, X-ray structural analysis, and porosity analysis. These papers focused, not on the material properties, but on new structures and their crystallographic topologies [48]. In category (2), properties related to gas adsorption, the studies targeted the separation of similar gases, energy storage, and understanding and designing the adsorption behavior [49]. Category (3), physical properties, includes studies on luminescence [50,51], magnetism [52], electron conductivities [53], and ion conductivities [54]. In most of the case, these functionalities can be facilitated from molecular components in MOFs. In category (4), catalysis, most of catalytic reactions are achieved by open metal sites [55] or chiral organic ligands [56]. Reactions of small organic molecules are the primary focus of these studies. Category (5), specific frameworks, includes in-depth studies of the famous structure with robust stability, along with tunability in systematic studies [57]. In category (6), the film and nanoparticles, there are numerous studies on nanocrystal and membrane fabrication [58]. In category (7), thermolysis, studies show how MOFs can be thermochemically converted to carbon-based materials or metal- and metal oxide-based materials [59]. In category (8), microwave and ionothermal synthesis, a synthetic method is adopted instead of hydrothermal synthesis, by which the rapid synthesis of new frameworks is possible [60]. In category (9), bioapplications, MOFs are used for drug delivery or cell cultivation [61]. In category (10), post synthesis metal/ligand exchange, new frameworks or compositions are studied [62].

### 3.4. Classification of Current Research Domain for Zeolites and MOFs

**Figure 6:**
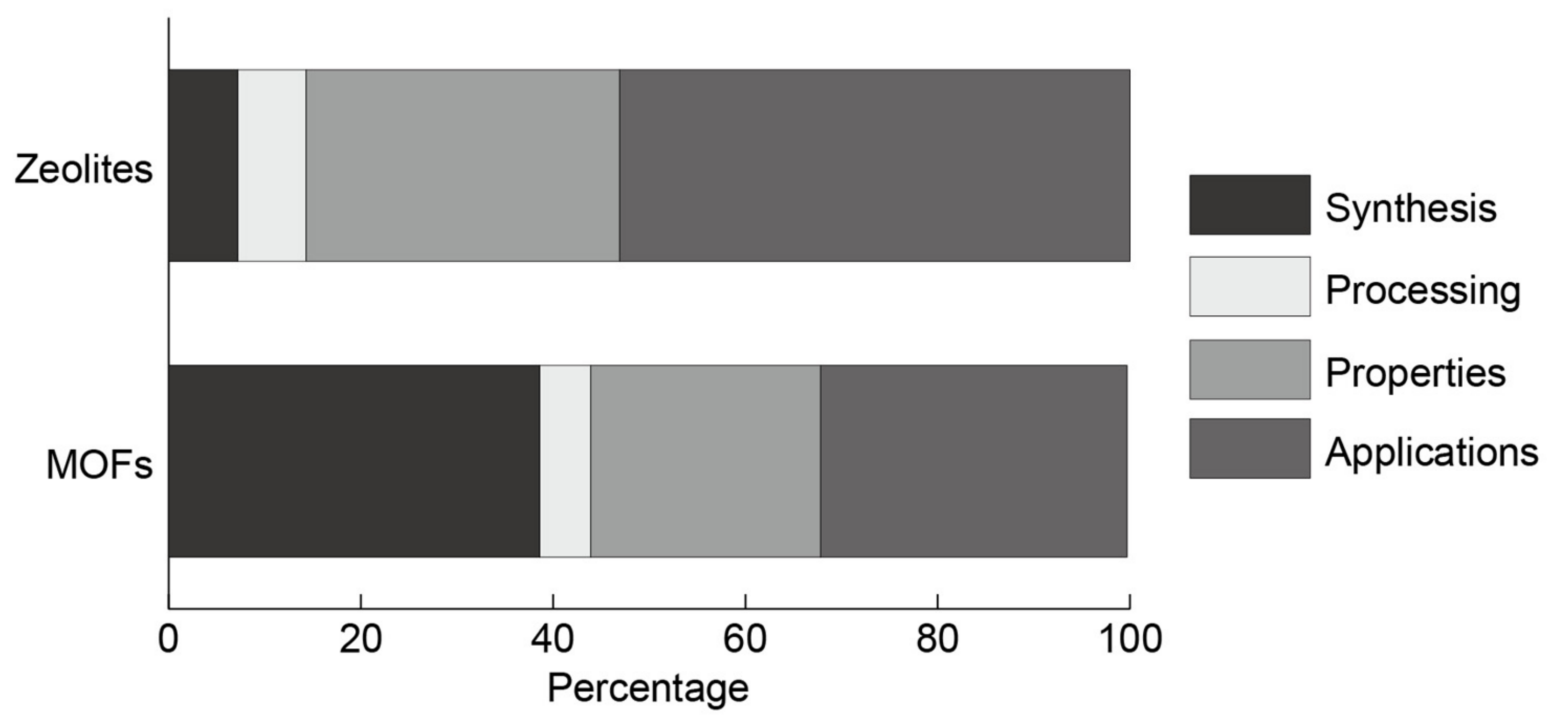
Ratios of publication numbers associated with four stages.

We next classify the research domain using the four stages defined in Scheme 2. Figure 6 summarizes the percentages and representative large domains for zeolites and MOFs. The research area for zeolites has been focused on “properties” and “applications”, whereas that for MOFs has been focused on “synthesis” and “applications”.

#### 3.4.1. Synthesis 

The synthesis stage proposes new type or new derivatives of MOF and zeolites.

##### Synthesis of Zeolites

Even though these materials are related, the approaches for synthesis were significantly different. In the case of zeolites, around 200 framework types of zeolites have been synthesized and reported [63]. Primarily, zeolites have been synthesized using hydrothermal methods, by which zeolite syntheses are carried out at high temperature under high pressure. The lower number of framework structures of zeolites is due to the low number of building units and mystery in the self-assembly processes of zeolites. In general, the synthesis of new framework structures is difficult to achieve. Therefore, other methods to control pore characteristics such as cation exchange and nanosheet fabrication have been developed [64]. Cation exchange is relatively easy and useful because an inorganic framework can be still stable during cation exchange. Morphology control is another research direction for zeolites. Nanoparticles, nanosheets, and other hierarchical pore structures are known to increase the effective surface area of zeolites and improve molecular diffusion for the catalysis. Controlling zeolite morphology is also related to processing, which will be discussed later.

##### Synthesis of MOFs

The synthesis of new MOFs is widely studied because of the variety in combinations of metal ions and bridging ligands. The coordination geometry around metal ions and connectivity through organic ligands play important roles in their crystal structure and porosity. These structures are important with regard to the surface area [65]. Likewise, the incorporation of functional building units basically allows their molecular functionality to be adopted by porous materials, as represented by chiral catalysis [66]. Most of these are synthesized using hydrothermal methods, along with microwave or ionothermal synthetic methods [67]. These frameworks are studied not only due to their properties or functions but due to their interest for topological structures, which are not found in inorganic salts or molecular crystals [68]. The complexity of their topological structure is also of interest to physicists in relation to the design of physical properties.

#### 3.4.2. Process 

The process stage focuses on efficient methods to synthesize in terms of cost, time and number of steps for synthesis.

##### Process of Zeolites

Processing in zeolite synthesis mainly includes two topics: (1) synthesizing zeolites from low-cost starting materials and (2) controlling zeolite morphologies. The starting materials for zeolites are relatively cheap, with a cost of approximately $200 to produce 1 kg of typical zeolite Y (see Supplementary Materials for the detailed calculation). For SiO_2_, using industrial wastes such as coal fly ash and asbestos has been well studied. The most expensive materials are template-like organic molecules called organic structure-directing agents (OSDAs). Recycling OSDAs [69] and the OSDA-free synthesis [70] of zeolites have been hot topics in recent years. The fabrication of zeolite membranes and films has been studied and industrially utilized [44]. The preparation of nanocrystals, nanosheets, and zeolites with hierarchical porosities has been reported using both top-down and bottom-up approaches [71].

##### Process of MOFs

Processing of MOFs is a minor topic in this research area. The chemicals needed for syntheses, such as organic ligands and solvents, are more expensive than the building blocks for zeolites. In case of MOF-5, which could be the most famous class of MOFs, this cost is more than $14,000 to produce 1 kg (see Supplementary Materials for the detailed calculation). This is because of the requirement for expensive organic solvents such as DMF and chloroform. Industrial researchers are still investigating the processing strategy. BASF started the electrolysis of Cu for the synthesis of HKUST-1 [Cu_3_(btc)_2_]*_n_* (btc = 1,3,5-benzenetricarboxylate) [72], and some researchers have started to use organic waste such as PET bottles as a route for MOF syntheses. [73] Solid-state synthesis can also be used as a green chemistry route [74]. However, organic ligands are still expensive from the chemical engineering point of view. These synthetic methods are also useful for the preparation of MOF films.

#### 3.4.3. Properties

The properties stage focuses on investigation of properties of synthesized materials.

##### Properties of Zeolites

One of the most important properties of zeolites is their acidity. Zeolites have Brønsted acidic proton sites as counterparts to anionic frameworks, which can be introduced by the isomorphous substitution of Si(IV) by Al(III) atoms in the TO_4/2_ (T = Si, Al) tetrahedral. These acidic sites are characterized using NH_3_ temperature programmed desorption [75] and solid-state nuclear magnetic resonance [76]. The Lewis acidity of zeolites induced by the isomorphous substitution of other metals such as Ti (IV) and Sn (IV) has recently been studied as well [77]. The thermal and hydrothermal stabilities of zeolites are well studied and depend on the structure types, framework compositions, and extra-framework cations.

##### Properties of MOFs

Adsorptive properties are tunable in terms of pore functionalization. MOF-74 [M_2_(dobdc)]*_n_* (dobdc = 2,5-dioxido-1,4-benzenedicarboxylate) can be synthesized with a variety of metal centers such as Zn^2+^, Ni^2+^, Mn^2+^, Co^2+^, and Fe^2+^. The H_2_ adsorption heat is also tunable [78]. These tunabilities in relation to the adsorption energy are particularly important for gas separation based on interactions [79]. The flexibility of the framework is specific for MOFs and has been shown as unique porous properties [80]. This is because such porous materials change their structures in response to guest adsorption, making these materials useful for some applications.

The properties of MOFs are versatile for various physicochemical applications. For example, cyano-based MOFs have been studied as porous magnets and guest-induced spin crossover behavior [81]. Multiferroic behavior was also observed for the perovskite type architecture [82]. Proton conductivities have been extensively studied by introducing proton carriers to pores [83,84]. Luminescent MOFs have been studied because of their excitation stability with the pore structure [50,51]. Semiconductor MOFs have recently been studied [85] and are expected to be used for applications that include electrocatalysis, photovoltaics, and solar cells.

MOFs are not generally thermally stable and calcined to carbon-based materials or metal oxide-based materials [86,87]. Pyrolysis can be used for the preparation of high-surface-area carbon materials, and aerobic oxidation is actually used for the preparation of metals or metal-oxide based materials. These are used for batteries and catalysis. Needless to mention, molecularly well-defined crystalline materials are transformed into disordered materials during pyrolysis, and it is difficult to understand the structure–property relationships.

#### 3.4.4. Applications

The applications stage focuses on the applications of a synthesized material for practical use.

##### Applications of Zeolites

Catalytic applications are more ideal for zeolites because of the use of robust frameworks and their acidic sites compared with MOFs. Common catalytic reactions utilize solid acid catalysts over zeolites. Acidic sites provide various organic reactivity such as cracking and isomerization [88]. Tons of zeolites have been used for the fluid catalytic cracking of heavy oils. Redox active sites are introduced using metal site substitutes such as Ti and Sn. The redox activity can be used for epoxidation, Baeyer–Villiger oxidation, and so on [89]. Extra-framework cations such as Cu^2+^ and encapsulated clusters such as Pt clusters can also be catalytic reaction sites. For example, the selective catalytic reduction of NO_x_ by Cu^2+^ has attracted attention for automotive uses. With respect to catalysis using zeolites, the rate-limiting step for the catalysis is sometimes diffusion-limited. Therefore, the nanostructuring of zeolites as hierarchical or sheet structures has been studied to improve the diffusion limitation [46].

##### Applications of MOFs

Gas adsorption applications as portable storage are more suitable for MOFs because of the light composition of formula weights. Hydrogen and methane gases are the general targets for gas storage as a demonstration of energy transport [90,91]. Because of their high surface area and strong interaction with guest molecules, they already exceed the theoretical limits for zeolites and carbon-based materials. Gas separation applications have also been studied. Syn-gas can be contaminated with CO_2_. Therefore, CO_2_/CH_4_ or C_2_H_6_/CH_4_ separation has been studied [92].

## 4. Discussion

### 4.1. “Well-Cited” Research Areas from Citation Network Analysis

We examined high impact areas of research by analyzing what types of research areas are acceptable to high-impact journals (HIJs) on average. First, HIJ indexes were calculated for the total publications on zeolites and MOFs by multiplying the impact factor by the number of publications and dividing by the total number of publications (see Equation (2) in methods and SI). In this analysis, the research areas for zeolites and MOFs were classified twice according to equation (1): each research area was clustered into subcategory A, B, C, etc. After being clustered, the category was segmented again into A-1, A-2, A-3, and so on in the same way. Zeolite A, B, C, D, and E represent cation exchange, basic characterization, catalysis, physical properties, and methane aromatization, respectively. On the other hand, MOF A, B, and C represent synthesis, gas adsorption, and new research directions, respectively. The HIJ index for whole MOF research areas was almost three-times that for zeolite research areas (HIJ index _MOF_ = 1.5 and HIJ index _zeolite_ = 0.60). The HIJ indexes and keywords for each cluster of zeolites and MOFs are listed in Table 1 and Table 2, respectively.

In the case of zeolites, papers on the fundamental characterization of the acidity and reaction mechanism were published by low impact factor journals as defined by the rest of the journals except for high impact journals. On the other hand, nanosheet fabrication, biomass production, and photo/electrocatalysts have been highlighted in HIJs. This is understandable from the viewpoint of applications. Zeolites are generally useful for catalysis, and more advanced research domains are highlighted.

In the case of MOFs, research articles on the random synthesis of new structures have been accepted by journals with relatively low impact factor. There are numerous papers on synthesis that is why these studies are rarely found in HIJs. On the other hand, papers on COFs and battery-related research for MOFs are accepted by HIJs. We think that this is related to the interest in energy technologies and, in the case of COFs, the interest shown by researchers in the fields of physical organic chemistry and polymer science. In both cases, papers on interdisciplinary science are accepted by HIJs.

### 4.2. Research Area Preferences by Country

**Figure 7:**
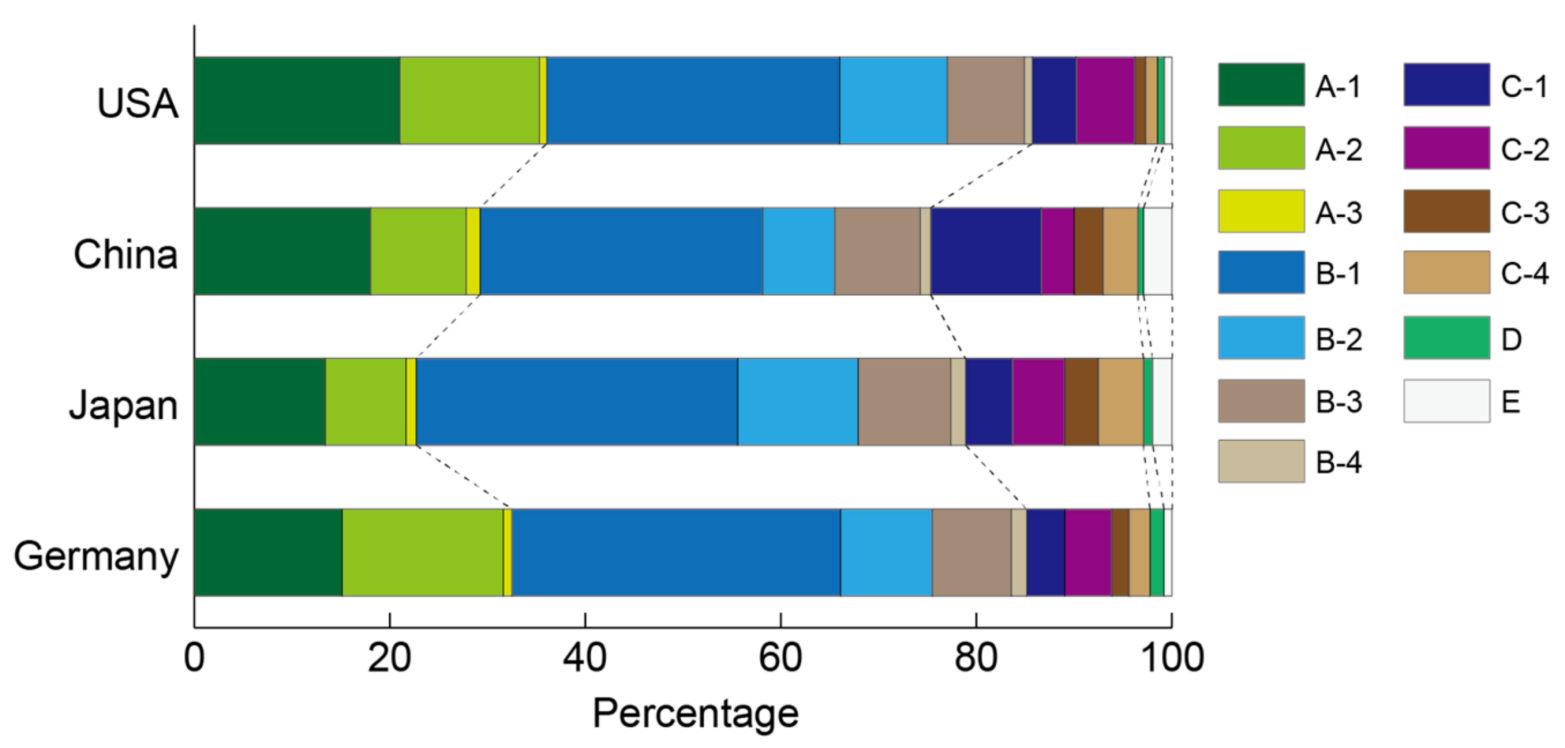
Research area population by representative countries for zeolites.

**Figure 8:**
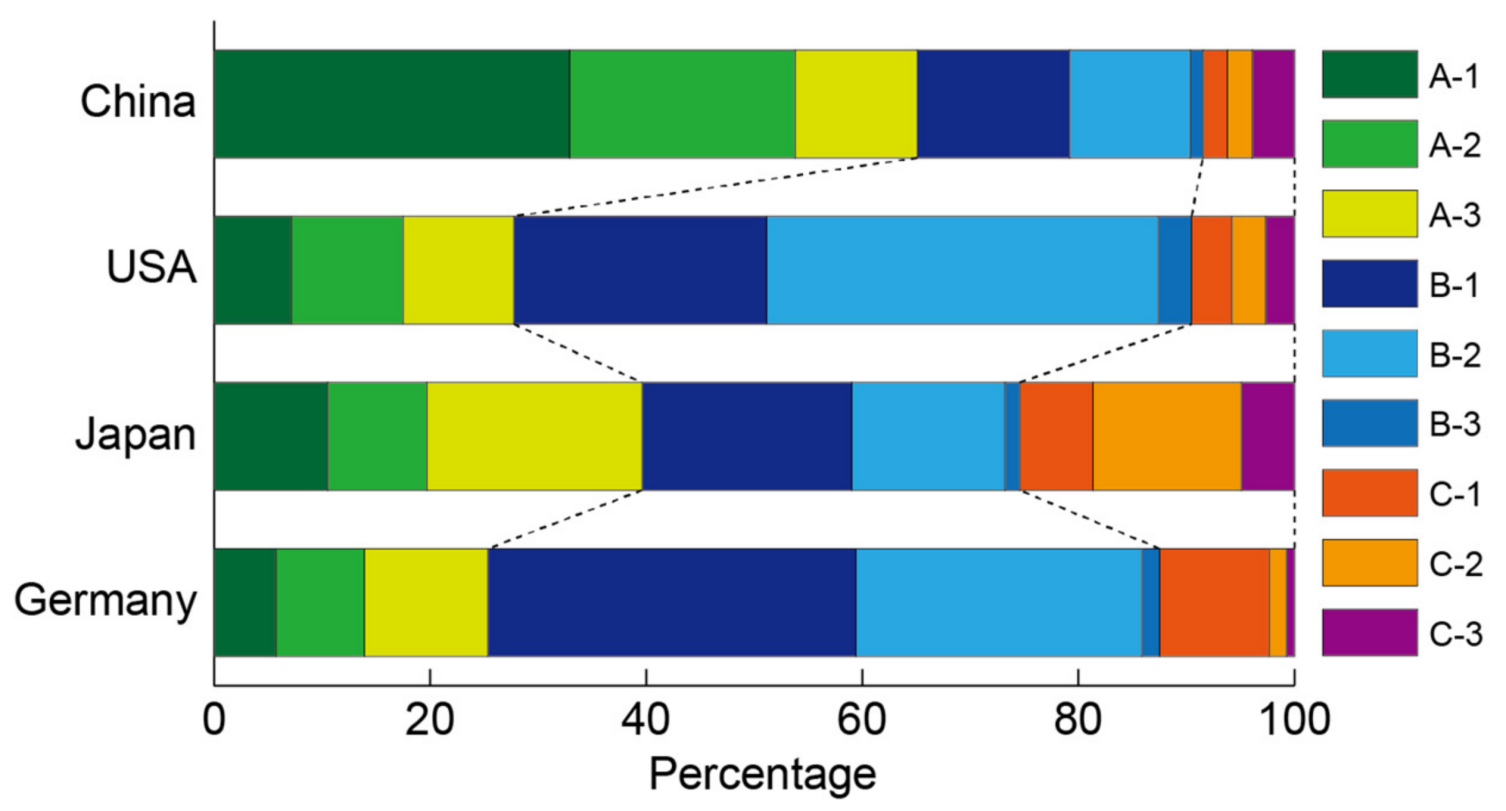
Research area population by representative countries for MOFs.

We also examined preferred research areas of representative countries (Figure 7 and Figure 8). China, USA, Japan, and Germany were selected in this case because 43% of publications for zeolites and 74% of publications for MOFs were published in these countries, as shown in Figure 2 and Figure 3. Interestingly, the research area largely depends on the country for both zeolites and MOFs.

In the case of zeolites, researchers in Germany develop zeolite-A-2 (membrane reactor). Chinese researchers mainly focus on nanosheet-based catalysis. USA researchers and Japanese researchers have advantages on cation exchange and the basic spectroscopic characterization of zeolites, respectively.

On the other hand, the research area heavily depends on the country for MOFs. Over 60% of Chinese researchers in this field studied the synthesis of MOFs. USA researchers mainly studied gas storage. German researchers studied catalysis, semiconductors, and films. Japanese researchers focused on the synthesis of MOFs and battery-based applications.

### 4.3. Why Are These Materials Suitable for the Current Research Area?

Currently, zeolites and MOFs are used for different applications. Considering the structure–property correlation is helpful to understand the current situation. Zeolites are robust due to the inorganic-based structures, and are therefore useful for the harsh conditions. Micropores of zeolites are usually smaller than 0.7 nm, which is suitable for molecular sieving. Moreover, isolated metal atoms incorporated into the silicate frameworks and/or cations introduced via ion-exchange show characteristic properties. On the other hand, MOFs are useful due to the designable structure, therefore, fundamental studies are currently ongoing based on X-ray crystallography and local modification of structure which can be used for minor modification for improvement of material properties. Future applications will be developed based on the discovery of useful materials and their processing with low synthetic costs.

Zeolites are mainly used for catalysis, adsorption, and ion-exchange. For example, cracking reactions have been used since the 1960s. In addition, on the laboratory scale, molecular sieves are used for solvent dehydration. The cation-exchange properties are suitable for detergent builders. Zeolites are also recently used for selective catalytic reduction of NOx in automotives, where zeolites are exposed to high temperature steaming conditions.

Regarding MOFs, (1) new structures; (2) gas adsorption; and (3) physical properties are extensively studied. Because of the various combinations of metal ions and bridging ligands, there is no synthetic limit on obtaining MOFs with new structures. In addition, their pore interactions and pore sizes are designable, which has prompted much research on gas adsorption. Their applications are not only limited for the porous properties but also applied for designer molecular solids. Some researchers started to use MOFs for mechanical stability, and as conducting materials.

### 4.4. Research Domain Having Potentials to Be Developed in the Future

This section discusses research clusters, which have been intensely studied in zeolites and MOFs, but are not the focus of other community. These research areas could potentially be developed in the future. Basically, the research areas classified as “application” in the zeolite area, such as harsh catalytic reactions, have not been examined in the MOF area. Similarly, the application areas for MOFs are seldom investigated in contrast to zeolites. This tendency seems to reflect the longer research history for zeolites compared to MOFs, with many studies in the “application” stage. Referring to the future applications of zeolites will be beneficial for MOFs. Here, we propose possible research areas for zeolites and MOFs, as listed in Table 3.

#### 4.4.1. From Zeolites to MOFs

First, well-studied research topics of zeolites, which have not been investigated as much in relation to MOFs, are listed as follows: membrane reactors; cation exchange, including radioactive decontamination and soil improvement use in agriculture; edible materials; the synthesis of zeolite nanosheets; and topotactic conversion methods. 

A membrane reactor is used for integrating catalytic reactions and separation processes (for example, xylene isomerization and *p*-xylene separation). Robust film preparation can be a primitive step for a membrane reactor. Zeolite films can be prepared using the secondary growth of deposited seed crystals. This research area is still small for zeolites, and it is hardly found for MOFs.

The cation exchange property is utilized in radioactive decontamination and soil improvement in zeolite research. Zeolites have metal oxide-based frameworks with exchangeable sites for cations. Cation exchange is one of the fascinating characteristics of zeolites. In MOF research, a few publications are found for the topic in ability to catch radioactive iodine, and no researcher has examined an application as a soil conditioner in agriculture. Compared with zeolites, cation exchange is generally more difficult because of the instability of MOFs. Cation exchanges have been reported for unstable frameworks such as MOF-5: [Zn_4_O(bdc)_3_]*_n_* (bdc = 1,4-benzenedicarboxylate) [93], and these studies will open up opportunities for single site catalysis.

Some zeolites are edible, and are harmless to animals. Edible MOFs have rarely been investigated by synthesizing MOFs from natural products [94]. Zeolite nanosheets with a single unit cell thickness (~2 nm) can be prepared using designed OSDAs. The bottom-up fabrication of zeolites from known layered silicates has been established through topotactic conversion. The top-down fabrication of layers using zeolites and reassembly processes has also been studied for germanosilicate zeolites and is known as the assembly-disassembly-organization-reassembly route.

As previously discussed, zeolites have a much longer research history and more topics focusing on applications in industry than MOFs. This difference results in the transfer of nine topics from zeolites to MOFs. All nine topics are related to actual applications.

#### 4.4.2. From MOFs to Zeolites

Physicochemical properties such as magnetism and the electronic conductivity of MOFs are also attractive aspects for the zeolites as well. Zeolites are metal oxide-based materials. Thus, they can exhibit certain physicochemical properties. Redox activity or work function tuning can be achieved using transition metal centers for tetrahedral sites. Even though the ligand field splitting for a tetrahedral site is large, an appropriate choice of metal ions can introduce sufficient super-exchange interaction through the oxides.

A flexible structural change in the framework is also potentially applicable to zeolites. In the case of MOFs, the framework structure is deformable through coordination bonds. This is applicable to zeolites by introducing deformable bonding or Jahn–Teller type distortion in the bonding. This holds promise for applications in guest-responsive sensors and catalysis.

As previously shown, MOFs have a much shorter research history and more topics focusing on synthesis and properties than zeolites. Zeolite research has been basically focused on catalysis and the cation exchange properties. Thus, other insights can contribute to further development of applications.

## 5. Conclusions

The research area of zeolites has been application-oriented compared with that on MOFs because of their solid acid and ion-exchange properties with robust frameworks. Designable zeolite synthesis based on the physical properties and framework structures will be a future challenge. On the other hand, research areas of MOFs are still being developed. Their structural designability leads to the control of physicochemical properties. Structural robustness of MOFs will be required for the future development of industrial applications and processes. At present, more applications can be found for zeolites than for MOFs.

The demonstrated methodology is not narrowly limited to the comparative review of zeolites and MOFs but is applicable to other comparative research areas. The demonstrated methodology enables (1) the rapid screening of related research areas; (2) classification of research stages in a domain; (3) investigation of the impact of a given research domain; (4) determination of the research area preferences of given countries and (5) proposing research area from zeolites to MOFs and vice versa. Moreover, the bibliometrics approach allows researchers to brainstorm and generate ideas on emerging research domains. Structured and objective knowledge makes it possible to get an overview of research domains, identify emerging domains, extract inter-domains, predict core-domains, and recommend future actions based on a meta-analysis of the research area.

## Figures and Tables

**Table 1:** High impact areas of research for zeolites.

Cluster	Publication Number	Average Year	HIJ Index	Keywords
A-1	13732	2003.5	0.71	Cation exchange
A-2	7000	2003.7	0.69	Film membrane
A-3	632	2006.7	1.05	Hierarchical pore, VOC removal
B-1	23381	2000.6	0.35	Characterization for acid sites
B-2	6789	2000.1	0.33	Reactions using extra-framework cations
B-3	6466	2000.1	0.23	Reactions using zeolite-supported metal clusters
B-4	1558	1992	0.17	Other reactions
C-1	3548	2008.4	0.88	Nanosheets, catalysis
C-2	3445	2002.7	1.12	Electron transfer, photo catalyst
C-3	2311	2004.2	0.18	Acid catalysis
C-4	1517	2005.9	0.56	TiO_2_-based photo catalysis
D	1250	1988.8	0.049	Physical properties
E	1096	2004.8	0.12	Methane aromatization

**Table 2:** High impact areas of research for MOFs.

Cluster	Publication Number	Average Year	HIJ Index	Keywords
A-1	4156	2011.4	0.42	Synthesis and new structure
A-2	3509	2012.3	1.17	Synthesis and new structure
A-3	2999	2009.6	1.07	Synthesis and new structure
B-1	4439	2013.2	2.36	Catalysis, semiconductor, film
B-2	4398	2012.3	2.12	Gas adsorption
B-3	419	2012.4	2.41	Energy storage related gas adsorption
C-1	698	2012.9	3.66	COF
C-2	694	2013.5	3.64	Battery, proton conductivity
C-3	685	2013.7	2.09	Thermolysis, magnetism

**Table 3:** Proposed research areas for zeolites and MOFs.

Zeolites to MOFs	MOFs to Zeolites
Membrane reactor	Basic physicochemical properties
Cation exchange (radioactive decontamination, soil improvement)	Flexible structure
Edible materials	
Nanosheets, topotactic conversion	

## Supplementary Materials

The following are available online at www.mdpi.com/1996-1944/10/12/1428/s1, Table S1: Effect of simulated impact factor for rest of journals, calculation of synthetic costs for zeolites and MOFs.

## References

[1] Breck D.W. (1974). Zeolite Molecular Sieves: Structure, Chemistry, and Use.

[2] Barrer R.M. (1948). 33. Synthesis of a zeolitic mineral with chabazite-like sorptive properties. J. Chem. Soc..

[3] Batten S.R., Champness N.R., Chen X.-M., Garcia-Martinez J., Kitagawa S., Ohrstrom L., O’Keeffe M., Suh M.P., Reedijk J. (2012). Coordination polymers, metal-organic frameworks and the need for terminology guidelines. CrystEngComm.

[4] Keggin J.F., Miles F.D. (1936). Structures and formulæ of the prussian blues andrelated compounds. Nature.

[5] Kondo M., Yoshitomi T., Matsuzaka H., Kitagawa S., Seki K. (1997). Three-dimensional framework with channeling cavities for small molecules: {[M_2_(4, 4′-bpy)_3_(NO_3_)_4_]·xH_2_O}_n_ (M = Co, Ni, Zn). Angew. Chem. Int. Ed..

[6] Kitagawa S., Kitaura R., Noro S.-I. (2004). Functional porous coordination polymers. Angew. Chem. Int. Ed..

[7] Ferey G. (2008). Hybrid porous solids: Past, present, future. Chem. Soc. Rev..

[8] Kajikawa Y., Abe K., Noda S. (2006). Filling the gap between researchers studying different materials and different methods: A proposal for structured keywords. J. Inf. Sci..

[9] Bar-Ilan J. (2008). Informetrics at the beginning of the 21st century—A review. J. Informetr..

[10] Rotolo D., Hicks D., Martin B.R. (2015). What is an emerging technology?. Res. Policy.

[11] Shibata N., Kajikawa A., Sakata I. (2011). Measuring relatedness between communities in a citation network. J. Am. Soc. Inf. Sci. Technol..

[12] Fujita K., Kajikawa Y., Mori J., Sakata I. (2014). Detecting research fronts using different types of weighted citation networks. J. Eng. Technol. Manag..

[13] Shibata N., Kajikawa Y., Takeda Y., Matsushima K. (2009). Comparative study on methods of detecting research fronts using different types of citation. J. Am. Soc. Inf. Sci. Technol..

[14] Ho J.C., Saw E.C., Lu L.Y.Y., Liu J.S. (2014). Technological barriers and research trends in fuel cell technologies: A citation network analysis. Technol. Forecast. Soc. Chang..

[15] Kajikawa Y., Takeda Y. (2009). Citation network analysis of organic LEDs. Technol. Forecast. Soc. Chang..

[16] Shibata N., Kajikawa Y., Takeda Y., Matsushima K. (2008). Detecting emerging research fronts based on topological measures in citation networks of scientific publications. Technovation.

[17] Takeda Y., Kajikawa Y. (2009). Optics: A bibliometric approach to detect emerging research domains and intellectual bases. Scientometrics.

[18] Glänzel W., Thijs B. (2012). Using ‘core documents’ for detecting and labelling new emerging topics. Scientometrics.

[19] Small H., Boyack K.W., Klavans R. (2014). Identifying emerging topics in science and technology. Res. Policy.

[20] Kajikawa Y., Takeda Y. (2008). Structure of research on biomass and bio-fuels: A citation-based approach. Technol. Forecast. Soc. Chang..

[21] Ogawa T., Kajikawa Y. (2015). Assessing the industrial opportunity of academic research with patent relatedness: A case study on polymer electrolyte fuel cells. Technol. Forecast. Soc. Chang..

[22] Nakamura H., Ii S., Chida H., Friedl K., Suzuki S., Mori J., Kajikawa Y. (2014). Shedding light on a neglected area: A new approach to knowledge creation. Sustain. Sci..

[23] Ittipanuvat V., Fujita K., Sakata I., Kajikawa Y. (2014). Finding linkage between technology and social issue: A literature based discovery approach. J. Eng. Technol. Manag..

[24] Shibata N., Kajikawa Y., Sakata I. (2010). Extracting the commercialization gap between science and technology case study of a solar cell. Technol. Forecast. Soc. Chang..

[25] Ogawa T., Kajikawa Y. (2017). Generating novel research ideas using computational intelligence: A case study involving fuel cells and ammonia synthesis. Technol. Forecast. Soc. Chang..

[26] Olson G.B. (1997). Computational design of hierarchically structured materials. Science.

[27] Olson G.B. (2000). Designing a new material world. Science.

[28] Yamaguchi Y., Komiyama H. (2001). Structuring knowledge project in nanotechnology materials program launched in japan. J. Nanopart. Res..

[29] Newman M.E.J., Girvan M. (2004). Finding and evaluating community structure in networks. Phys. Rev. E.

[30] Newman M. (2004). Fast algorithm for detecting community structure in networks. Phys. Rev. E.

[31] Kajikawa Y., Ohno J., Takeda Y., Matsushima K., Komiyama H. (2007). Creating an academic landscape of sustainability science: An analysis of the citation netwsork. Sustain. Sci..

[32] Takeda Y., Kajikawa Y. (2010). Tracking modularity in citation networks. Scientometrics.

[33] Corma A. (2003). State of the art and future challenges of zeolites as catalysts. J. Catal..

[34] Čejka J., Centi G., Perez-Pariente J., Roth W.J. (2012). Zeolite-based materials for novel catalytic applications: Opportunities, perspectives and open problems. Catal. Today.

[35] Bordiga S., Lamberti C., Bonino F., Travert A., Thibault-Starzyk F. (2015). Probing zeolites by vibrational spectroscopies. Chem. Soc. Rev..

[36] Groen J.C., Peffer L.A.A., Pérez-Ramírez J. (2003). Pore size determination in modified micro- and mesoporous materials. Pitfalls and limitations in gas adsorption data analysis. Microporous Mesoporous Mater..

[37] Smit B., Maesen T.L.M. (2008). Towards a molecular understanding of shape selectivity. Nature.

[38] Khan F.I., Ghoshal A.K. (2000). Removal of volatile organic compounds from polluted air. J. Loss Prev. Process Ind..

[39] Hedström A. (2001). Ion exchange of ammonium in zeolites: A literature review. J. Environ. Eng..

[40] Simon U., Franke M.E. (2000). Electrical properties of nanoscaled host/guest compounds. Microporous Mesoporous Mater..

[41] Cundy C.S., Cox P.A. (2003). The hydrothermal synthesis of zeolites: History and development from the earliest days to the present time. Chem. Rev..

[42] Davis M.E. (2014). Zeolites from a materials chemistry perspective. Chem. Mater..

[43] Caro J., Noack M. (2008). Zeolite membranes—Recent developments and progress. Microporous Mesoporous Mater..

[44] Rangnekar N., Mittal N., Elyassi B., Caro J., Tsapatsis M. (2015). Zeolite membranes—A review and comparison with mofs. Chem. Soc. Rev..

[45] Gates B.C. (1995). Supported metal clusters: Synthesis, structure, and catalysis. Chem. Rev..

[46] Perez-Ramirez J., Christensen C.H., Egeblad K., Christensen C.H., Groen J.C. (2008). Hierarchical zeolites: Enhanced utilisation of microporous crystals in catalysis by advances in materials design. Chem. Soc. Rev..

[47] Yaghi O.M., O’Keeffe M., Ockwig N.W., Chae H.K., Eddaoudi M., Kim J. (2003). Reticular synthesis and the design of new materials. Nature.

[48] Ockwig N.W., Delgado-Friedrichs O., O’Keeffe M., Yaghi O.M. (2005). Reticular chemistry: Occurrence and taxonomy of nets and grammar for the design of frameworks. Acc. Chem. Res..

[49] Li J.-R., Sculley J., Zhou H.-C. (2012). Metal-organic frameworks for separations. Chem. Rev..

[50] Allendorf M.D., Bauer C.A., Bhakta R.K., Houk R.J.T. (2009). Luminescent metal-organic frameworks. Chem. Soc. Rev..

[51] Cui Y., Yue Y., Qian G., Chen B. (2012). Luminescent functional metal-organic frameworks. Chem. Rev..

[52] Kurmoo M. (2009). Magnetic metal-organic frameworks. Chem. Soc. Rev..

[53] Sun L., Campbell M.G., Dincă M. (2016). Electrically conductive porous metal–organic frameworks. Angew. Chem. Int. Ed..

[54] Horike S., Umeyama D., Kitagawa S. (2013). Ion conductivity and transport by porous coordination polymers and metal–organic frameworks. Acc. Chem. Res..

[55] Lee J., Farha O.K., Roberts J., Scheidt K.A., Nguyen S.T., Hupp J.T. (2009). Metal-organic framework materials as catalysts. Chem. Soc. Rev..

[56] Ma L., Abney C., Lin W. (2009). Enantioselective catalysis with homochiral metal-organic frameworks. Chem. Soc. Rev..

[57] Park K.S., Ni Z., Côté A.P., Choi J.Y., Huang R., Uribe-Romo F.J., Chae H.K., O’Keeffe M., Yaghi O.M. (2006). Exceptional chemical and thermal stability of zeolitic imidazolate frameworks. Proc. Natl. Acad. Sci. USA.

[58] Furukawa S., Reboul J., Diring S., Sumida K., Kitagawa S. (2014). Structuring of metal-organic frameworks at the mesoscopic/macroscopic scale. Chem. Soc. Rev..

[59] Sun J.-K., Xu Q. (2014). Functional materials derived from open framework templates/precursors: Synthesis and applications. Energy Environ. Sci..

[60] Parnham E.R., Morris R.E. (2007). Ionothermal synthesis of zeolites, metal–organic frameworks, and inorganic–organic hybrids. Acc. Chem. Res..

[61] Giménez-Marqués M., Hidalgo T., Serre C., Horcajada P. (2016). Nanostructured metal-organic frameworks and their bio-related applications. Coord. Chem. Rev..

[62] Wang Z., Cohen S.M. (2009). Postsynthetic modification of metal-organic frameworks. Chem. Soc. Rev..

[63] Report I. IZA Structure Database. http://www.iza-structure.org/databases/.

[64] Choi M., Na K., Kim J., Sakamoto Y., Terasaki O., Ryoo R. (2009). Stable single-unit-cell nanosheets of zeolite mfi as active and long-lived catalysts. Nature.

[65] Chae H.K., Siberio-Perez D.Y., Kim J., Go Y., Eddaoudi M., Matzger A.J., O’Keeffe M., Yaghi O.M. (2004). A route to high surface area, porosity and inclusion of large molecules in crystals. Nature.

[66] Seo J.S., Whang D., Lee H., Jun S.I., Oh J., Jeon Y.J., Kim K. (2000). A homochiral metal-organic porous material for enantioselective separation and catalysis. Nature.

[67] Norbert Stock S.B. (2011). Synthesis of metal-organic frameworks (MOFs): Routes to various MOF topologies, morphologies, and composites. Chem. Rev..

[68] Batten S.R., Robson R. (1998). Interpenetrating nets: Ordered, periodic entanglement. Angew. Chem. Int. Ed..

[69] Lee H., Zones S.I., Davis M.E. (2003). A combustion-free methodology for synthesizing zeolites and zeolite-like materials. Nature.

[70] Iyoki K., Itabashi K., Okubo T. (2014). Progress in seed-assisted synthesis of zeolites without using organic structure-directing agents. Microporous Mesoporous Mater..

[71] Valtchev V., Tosheva L. (2013). Porous nanosized particles: Preparation, properties, and applications. Chem. Rev..

[72] Mueller U., Schubert M., Teich F., Puetter H., Schierle-Arndt K., Pastre J. (2006). Metal-organic frameworks—Prospective industrial applications. J. Mater. Chem..

[73] Deleu W.P.R., Stassen I., Jonckheere D., Ameloot R., De Vos D.E. (2016). Waste pet (bottles) as a resource or substrate for mof synthesis. J. Mater. Chem. A.

[74] James S.L., Adams C.J., Bolm C., Braga D., Collier P., Friscic T., Grepioni F., Harris K.D.M., Hyett G., Jones W. (2012). Mechanochemistry: Opportunities for new and cleaner synthesis. Chem. Soc. Rev..

[75] Niwa M., Katada N. (2013). New method for the temperature- programmed desorption (TPD) of ammonia experiment for characterization of zeolite acidity: A review. Chem. Rec..

[76] Feng N.D., Zheng A.M., Huang S.J., Zhang H.L., Yu N.Y., Yang C.Y., Liu S.B., Deng F. (2010). Combined solid-state NMR and theoretical calculation studies of bronsted acid properties in anhydrous 12-molybdophosphoric acid. J. Phys. Chem. C.

[77] Gunther W.R., Michaelis V.K., Griffin R.G., Roman-Leshkov Y. (2016). Interrogating the lewis acidity of metal sites in beta zeolites with ^15^N pyridine adsorption coupled with MAS NMR spectroscopy. J. Phys. Chem. C.

[78] Caskey S.R., Wong-Foy A.G., Matzger A.J. (2008). Dramatic tuning of carbon dioxide uptake via metal substitution in a coordination polymer with cylindrical pores. J. Am. Chem. Soc..

[79] Li J.-R., Kuppler R.J., Zhou H.-C. (2009). Selective gas adsorption and separation in metal-organic frameworks. Chem. Soc. Rev..

[80] Horike S., Shimomura S., Kitagawa S. (2009). Soft porous crystals. Nat. Chem..

[81] Southon P.D., Liu L., Fellows E.A., Price D.J., Halder G.J., Chapman K.W., Moubaraki B., Murray K.S., Letard J.F., Kepert C.J. (2009). Dynamic interplay between spin-crossover and host-guest function in a nanoporous metal-organic framework material. J. Am. Chem. Soc..

[82] Jain P., Ramachandran V., Clark R.J., Zhou H.D., Toby B.H., Dalal N.S., Kroto H.W., Cheetham A.K. (2009). Multiferroic behavior associated with an order-disorder hydrogen bonding transition in metal-organic frameworks (MOFs) with the perovskite ABX(3) architecture. J. Am. Chem. Soc..

[83] Bureekaew S., Horike S., Higuchi M., Mizuno M., Kawamura T., Tanaka D., Yanai N., Kitagawa S. (2009). One-dimensional imidazole aggregate in aluminium porous coordination polymers with high proton conductivity. Nat. Mater..

[84] Hurd J.A., Vaidhyanathan R., Thangadurai V., Ratcliffe C.I., Moudrakovski I.L., Shimizu G.K.H. (2009). Anhydrous proton conduction at 150 °C in a crystalline metal–organic framework. Nat. Chem..

[85] Hendon C.H., Tiana D., Walsh A. (2012). Conductive metal-organic frameworks and networks: Fact or fantasy?. Phys. Chem. Chem. Phys..

[86] Liu B., Shioyama H., Akita T., Xu Q. (2008). Metal-organic framework as a template for porous carbon synthesis. J. Am. Chem. Soc..

[87] Hu M., Reboul J., Furukawa S., Torad N.L., Ji Q., Srinivasu P., Ariga K., Kitagawa S., Yamauchi Y. (2012). Direct carbonization of al-based porous coordination polymer for synthesis of nanoporous carbon. J. Am. Chem. Soc..

[88] Yilmaz B., Muller U. (2009). Catalytic applications of zeolites in chemical industry. Top. Catal..

[89] Corma A., Nemeth L.T., Renz M., Valencia S. (2001). Sn-zeolite beta as a heterogeneous chemoselective catalyst for baeyer-villiger oxidations. Nature.

[90] Li Y.W., Yang R.T. (2006). Hydrogen storage in metal-organic frameworks by bridged hydrogen spillover. J. Am. Chem. Soc..

[91] Eddaoudi M., Kim J., Rosi N., Vodak D., Wachter J., O’Keeffe M., Yaghi O.M. (2002). Systematic design of pore size and functionality in isoreticular MOFs and their application in methane storage. Science.

[92] Sumida K., Rogow D.L., Mason J.A., McDonald T.M., Bloch E.D., Herm Z.R., Bae T.H., Long J.R. (2012). Carbon dioxide capture in metal-organic frameworks. Chem. Rev..

[93] Brozek C.K., Dinca M. (2014). Cation exchange at the secondary building units of metal-organic frameworks. Chem. Soc. Rev..

[94] Smaldone R.A., Forgan R.S., Furukawa H., Gassensmith J.J., Slawin A.M.Z., Yaghi O.M., Stoddart J.F. (2010). Metal-organic frameworks from edible natural products. Angew. Chem. Int. Ed..

