# Supplementary material for: Landscape of Research Areas for Zeolites and Metal-Organic Frameworks Using Computational Classification Based on Citation Networks
Source: Materials (Basel). 2017 Dec 14;10(12):1428. doi: 10.3390/ma10121428 (PMC5744363; doi:10.3390/ma10121428)
Supplement: Supplementary file 1 [file materials-10-01428-s001.pdf]

Supplementary Materials

Landscape of Research Areas for Zeolites and Metal-organic Frameworks: Computational Detection of Emerging Research Domains

Takaya Ogawa[1,*,†,‖], Kenta Iyoki[2,*,‡,‖], Tomohiro Fukushima[1,*,§,‖] and Yuya Kajikawa[3]

[1]Department of Chemistry, Massachusetts Institute of Technology, 77 Massachusetts Avenue, Cambridge, MA 02139, USA

[2]Department of Chemical Engineering, Massachusetts Institute of Technology, 77 Massachusetts Avenue, Cambridge, MA 02139, USA

[3]School of Environment and Society, Tokyo Institute of Technology, 3-3-6 Shibaura, Minato-ku, Tokyo 108-0023, Japan; kajikawa@mot.titech.ac.jp

[*]Correspondence: takaya@mit.edu or t.o.1221@gmail.com (T.O.); k_iyoki@chemsys.t.u-tokyo.ac.jp or iyoki.kenta@gmail.com (K.I.); tfuku@sci.hokudai.ac.jp or f.ky.note@gmail.com (T.F.)

[†]SUNCAT Center for Interface Science and Catalysis, Department of Chemical Engineering Stanford University, 443 Via Ortega, Stanford, CA 94305, USA.

[‡]Department of Chemical System Engineering, The University of Tokyo, 7-3-1 Hongo, Bunkyo-ku, Tokyo 113-8656, Japan.

[§]Department of Chemistry, Faculty of Science, Hokkaido University, Sapporo, Hokkaido 060-0810, Japan.

*E-mail:
takaya@mit.edu, t.o.1221@gmail.com (TO),
k_iyoki@chemsys.t.u-tokyo.ac.jp, iyoki.kenta@gmail.com (KI),
tfuku@sci.hokudai.ac.jp, f.ky.note@gmail.com (TF)

Table of Contents

**Contents**	**Pages**
Note for high impact journal (HIJ) indexes	S3
Journal distribution of publication number for zeolites and MOFs	S4-6
Calculation of synthetic costs for zeolites and MOFs	S7-S8

Note for high impact journal (HIJ) indexes

In this analysis, we focused on the high impact journal for given cluster. High impact journals are counted and averaged with number of publication whereas low impact journals are neglected in the contribution.

Table S1 shows the effect of simulated impact factor for rest of journals in the report. We varied impact factor from 0 to 4 for the rest of journals but relative tendency between the cluster was found to be similar to each impact factor.

$$High\ impact\ journal\ index\ of\ a\ cluster = \frac{\sum n_i \times IF_j + (n_{total} - \sum n_i) \times IF_x}{n_{total}}$$

eqn(2)

Table S1. Effect of simulated impact factor for rest of journals

#	IFx = 0	IFx = 2	IFx = 4	IFx = 6	IFx = 8
Zeolite-1	0.882	2.758	4.634	6.510	8.386
Zeolite-2	0.350	2.293	4.236	6.179	8.121
Zeolite-3	0.699	2.584	4.470	6.356	8.241
Zeolite-4	0.0488	2.041	4.033	6.025	8.017
Zeolite-5	0.125	2.103	4.081	6.059	8.037
MOF-1	0.827	2.668	4.508	6.349	8.189
MOF-2	2.239	3.871	5.503	7.135	8.767
MOF-3	2.960	4.463	5.965	7.467	8.969

Table S2. Journal distribution of publication number for MOFs

	A-1	A-2	A-3	B-1	B-2	B-3	C-1	C-2	C-3
Nature	1	2	2	5	6	0	1	1	0
Nature Chem	3	2	2	12	15	4	10	8	0
Nature Mater	1	3	3	14	4	2	0	2	0
Nature Comm	1	4	1	20	22	2	9	5	7
Science	1	1	4	11	13	0	4	2	0
PNAS	1	1	2	5	8	0	1	1	2
JACS	39	104	78	240	225	27	51	70	24
Angew	31	66	79	154	104	8	43	35	21
Chem Sci	3	17	6	47	36	4	11	10	7
EES	1	0	1	17	49	7	1	6	6
Adv Mater	2	9	2	43	24	2	14	7	11
Chem Commun	87	202	114	327	241	15	66	54	53
Total	4156	3509	2999	4439	4398	419	698	694	685

Table S3-1. Journal distribution of publication number for Zeolites

	A-1	A-2	A-3	A-4	B-1	B-2	B-3	B-4	
Nature	56	8	13	1	20	2	7	1	
Nature Chem	7	3	6	0	1	0	0	0	
Nature Mater	15	5	6	1	2	1	0	0	
Nature Comm	5	4	9	0	3	1	1	0	
Science	26	15	10	2	8	1	2	0	
PNAS	10	4	5	0	3	1	0	0	
JACS	210	103	176	6	324	92	50	6	
Angew	166	72	117	12	119	27	24	6	
Chem Sci	9	1	16	2	9	3	2	2	
EES	1	9	37	1	3	1	0	0	
Adv Mater	13	42	20	10	9	3	1	1	
Chem Commun	137	70	164	11	171	65	28	6	
Total	13732	7000	4421	632	23381	6789	6466	1558	

Table S3-2. Journal distribution of publication number for Zeolites

	C-1	C-2	C-3	C-4	D	E
Nature	8	6	1	2	0	0
Nature Chem	3	0	0	0	0	0
Nature Mater	3	1	0	0	0	0
Nature Comm	4	0	0	0	0	0
Science	5	6	0	1	0	1
PNAS	4	1	0	3	1	0
JACS	47	131	2	26	2	4
Angew	67	42	9	11	0	1
Chem Sci	4	4	3	0	0	0
EES	6	2	0	0	0	0
Adv Mater	7	28	1	8	1	0
Chem Commun	96	85	29	14	1	6
Total	3548	3445	2311	1517	1250	1096

Calculation of synthetic costs for zeolites and MOFs

Synthesis of NaY zeolites in 1 kg scale. 200 dollar

#	Aldrich Number	Unit price/dollar	Required amount	Price/dollar
NaOH pellet	221465-50KG	1908	47.4 g	1.8
Sodium aluminate	13404-5KG-R	237	431 g	20.4
Sodium silicate solution	338443-3L	167	4.7 kg	187.8

Batch Preparation (for 32 g anhydrous product)

Seed Gel

(1) [19.95 g water + 4.07 g sodium hydroxide + 2.09 g sodium aluminate], stir in 50 mL plastic bottle until dissolved

(2) [(1) + 22.72 g sodium silicate solution], stir moderately for at least 10 minutes; after stirring, cap the bottle and let the solution age at room temperature for 1 day

Feedstock Gel

(3) [130.97 g water + 0.14 g sodium hydroxide + 13.09 g sodium aluminate], stir in a 500 mL plastic beaker until dissolved

(4) [(3) + 142.43 g sodium silicate solution], stir vigorously with a high-shear turbine mixer until the gel appears somewhat smooth; cover the beaker until the addition of the seed gel

Overall Gel

(5) [(4) + 16.50 g of (2)], slowly add seed gel (2) to feedstock gel (4) under high shear; move the beaker during mixing to ensure the entire gel volume encounters the high shear from the turbine (up to 20 minutes)

Crystallization

Vessel: 300 mL polypropylene bottle (sealed)

Incubation: One day at room temperature

Temperature: 100°C

Time: After about 5 h, the gel will separate into a solid (containing the NaY Zeolite) that

will settle to the bottom, and a hazy supernatant liquid. Continue heating until the supernatant is clear indicating complete crystallization (no more than 2 additional hours)

Verified syntheses of zeolitic materials, 3rd revised edition, S. Mintova ed. ISBN: 978-0-692-68539-6

Synthesis of MOF-5 in 1kg scale. 14k dollar.

#	Aldrich Number	Unite price/dollar	Required amount	Price/dollar
$Zn(OAc)_2H_2O$	Z0625-1KG	77.20	3.4kg	262.48
Terephthalic acid	185361-500G	29.90	1kg	59.80
Triethylamine	T0886-1L	100.00	1.7litter	170.00
DMF	227056-18L	790	230litter	10094.44
$CHCl_3$	650498-4X4L	825	70 litter	3609.375

"*Terephthalic acid (5.065 g, 30.5 mmol) and triethylamine (8.5 mL) were dissolved in 400 mL of DMF. $Zn(OAc)_2{\cdot}2H_2O$ (16.99 g, 77.4 mmol) was dissolved in 500 mL of DMF. The zinc salt solution was added to the organic solution with stirring over 15 min, forming a precipitate, and the mixture was stirred for 2.5 h. A sample of the mixture, still damp, was used for PXRD analysis, which showed pure MOF-5 by comparison with the pattern simulated from SXRD data (Fig. 2a).[2] The precipitate was filtered and immersed in DMF (250 mL) overnight. It was then filtered again and immersed in $CHCl_3$ (350 mL, HPLC grade). The solvent was exchanged 3 times over 7 days: after 2 days, 3 days, and 7 days. The bulk of the solvent was decanted and the product was evacuated overnight to a pressure of 10 mTorr. It was activated at 120 °C and 10 mTorr for 6 h, at which point it was transferred to a glovebox and weighed (4.92 g, 63%).*"

Reference

http://dx.doi.org/10.1016/j.tet.2008.06.036
